# Replication of clinical prosthetic sockets for research purposes

**DOI:** 10.1097/PXR.0000000000000386

**Published:** 2024-11-27

**Authors:** Alix Chadwell, Laurence Kenney, Michael Prince, Jennifer Olsen, Matthew Dyson

**Affiliations:** 1 School of Healthcare Enterprise and Innovation, Faculty of Medicine, University of Southampton, Southampton, UK; 2 Centre for Human Movement and Rehabilitation, School of Health and Society, University of Salford, Salford, UK; 3 School of Engineering, Newcastle University, Newcastle upon Tyne, UK

**Keywords:** prosthetic socket, upper limb, replication, cloning, validation

## Abstract

For research in the field of prosthetics to be representative of clinical realities, studies require inclusion of clinical standard prosthetic sockets. This necessitates involvement of a prosthetist (clinical professional) in any study, which is to truly explore the effectiveness of existing or novel prosthetic technologies. Unfortunately, there is a global shortage of prosthetists. With many technological advances in upper-limb prosthetics coming from engineering focused labs, it is unsurprising that studies are frequently conducted with anatomically intact individuals. In this paper, we present a method to clone the shape of a clinical standard prosthetic socket for research purposes. The technique uses silicone to capture the socket shape; this is then converted into a plaster mold, which can be used to manufacture an identically shaped socket using standard clinical manufacturing techniques. The whole process can be achieved without the involvement of a prosthetist. To validate the proposed technique, molds from an original socket and socket clone were 3D scanned. The distance between the aligned meshes were measured using CloudCompare software. The mean distance between the points on the 2 meshes was 0.16 mm (standard deviation 0.38 mm). This proof-of-concept study demonstrates that the proposed new approach to cloning a clinical standard prosthetic socket is feasible and accurate. This technique will facilitate improvements in the assessment of prosthetic technologies. The process is nondestructive, thus also opening opportunities for socket design and electrode placement research with the removal of confounding factors relating to socket shape.

## Introduction

Research into upper-limb prosthetics is often limited by access to prosthetic sockets, each custom-fitted by a prosthetist. Socket design and manufacture is rarely documented.^1^ Studies requiring socket manufacture are often small, recruiting only people local to the research team/clinic. Manufacture of bespoke sockets on a larger scale is costly and puts excessive strain on an already stretched clinical service.^2–5^ Without an alternative feasible and accurate approach to socket creation for research purposes, many technical initiatives such as take-home training, real-world monitoring, and large-scale pattern recognition studies remain challenging to deliver. This paper presents a proof-of-concept for a process to clone a participant's existing socket shape.

Physical replication techniques are sometimes used clinically^6^; however, in all cases we could identify, the replication process is poorly documented, sometimes destructive, and mold removal can be difficult with narrow or long upper-limb sockets. We were also unable to identify any validation studies of such approaches. Photogrammetry, to digitally replicate internal socket surfaces, has been shown to have some success for lower-limb sockets^7^; however, as an upper-limb socket is smaller and usually bent at the elbow, line-of-sight can be obstructed.

A reliable and accurate socket replication process would have wider impacts beyond simply enabling research studies to reflect clinical realities. In the absence of a validated socket replication method, controlled studies of the impact of socket shape and physical attributes, such as mechanical compliance, on fit and comfort are difficult to deliver, as the nature of casting means that no 2 sockets manufactured for a person will be the same.

In this paper, we demonstrate the feasibility of accurately cloning a prosthetic socket's shape without damaging the original. A case study is presented, demonstrating the accuracy with which both the shape and precise electrode position for a myoelectric socket can be captured.

## Methods

A mold of the socket was taken using Platsil Gel silicone. Equivalent silicone can be used with shore hardness ranging from 00-30 (Platsil00) to A-60 (Platsil25). We found softer silicone easier to demold. Table 1 summarizes the proposed technique.

**Table 1.**
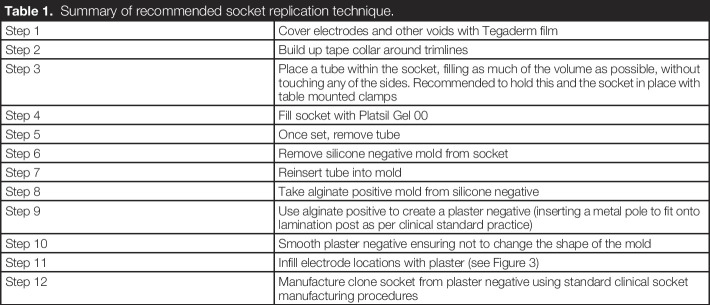
Summary of recommended socket replication technique.

Step 1	Cover electrodes and other voids with Tegaderm film
Step 2	Build up tape collar around trimlines
Step 3	Place a tube within the socket, filling as much of the volume as possible, without touching any of the sides. Recommended to hold this and the socket in place with table mounted clamps
Step 4	Fill socket with Platsil Gel 00
Step 5	Once set, remove tube
Step 6	Remove silicone negative mold from socket
Step 7	Reinsert tube into mold
Step 8	Take alginate positive mold from silicone negative
Step 9	Use alginate positive to create a plaster negative (inserting a metal pole to fit onto lamination post as per clinical standard practice)
Step 10	Smooth plaster negative ensuring not to change the shape of the mold
Step 11	Infill electrode locations with plaster (see Figure 3)
Step 12	Manufacture clone socket from plaster negative using standard clinical socket manufacturing procedures

To help guide future researchers, we have included supplementary information on the techniques we chose not to take forward (and why). These included the use of alginate and Limbtex Limbcopy silicone, and different approaches to mold capture where the socket was filled completely with silicone (“full-fill”) or lined with a thin layer (“layer coating”). See document, Supplemental Digital Content 1, http://links.lww.com/POI/A265.

### Socket preparation

To prepare the socket and avoid leakage of molding agent, the electrodes and any holes were covered with Tegaderm film bandage. Tegaderm is thin and elastic and adheres well without leaving residue, conforming to the electrodes without significantly impacting the socket surface. A tape collar was also built up above the socket trim lines.

### Socket shape capture

Our method combines the compressibility and easy extraction of layer coating with the structural benefits of full-fill. Before filling the socket with silicone, we placed a tube into the socket (ensuring it did not touch the edges); this was supported by a desk-mounted clamp while the silicone set. The tube diameter was chosen to fill as much of the socket as feasible so that once removed, the mold could be compressed and displaced from the socket walls. The tube could then be reinserted into the mold after extraction to ensure the shape was maintained. The tube was covered with clingfilm to ease extraction.

### Conversion of silicone mold to plaster mold

**Figure 1.**
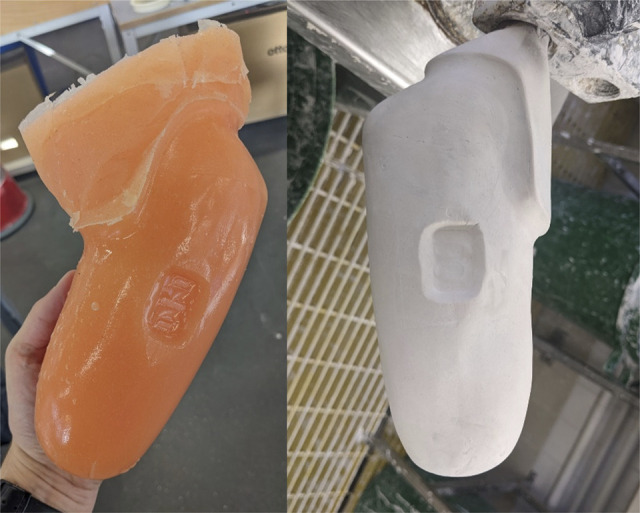
Left: Silicone negative mold produced from original socket; right: smoothed plaster negative mold produced from silicone negative via an alginate positive.

The silicone negative mold was converted to plaster (Figure 1) via an alginate positive. As there is risk of compound error, it was important to carefully avoid changes in the mold shape between steps. The plaster negative was carefully smoothed to remove bumps from bubbles in the alginate while avoiding changes to the shape or volume. The area above the trim lines was tidied to prevent the bag tearing during lamination.

### Electrode infill

**Figure 2.**
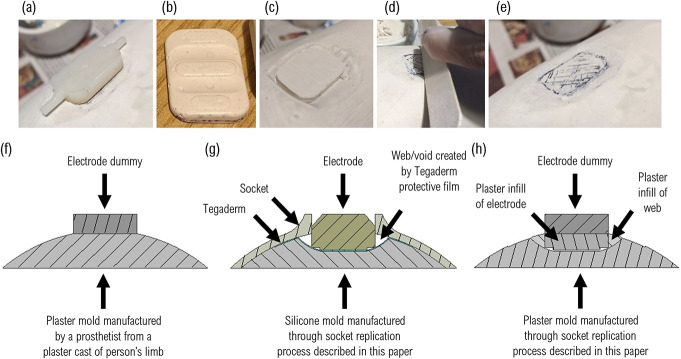
(a) An electrode dummy sits flush against a socket. (b-e) A plaster electrode infill, shaped to match the front of the electrode, and at a thickness which when combined with the thickness of the dummy, matches the electrode. This is placed into the recess in the socket, seated in the correct location by the shaping from the metal electrode contacts and colored in to mark its position. The void around the edge is filled with wet plaster and flattened so as not to adjust the shape of the socket itself. (f-h) Cross-sectional image showing how the electrode dummy would sit against the prosthetist's rectified mold (f) vs how the electrode infill would operate to replicate this for the clone socket (h).

Figure 1 shows the impression the electrodes left in the mold. When traditionally manufacturing a myoelectric socket, electrode dummies are used (Figure 2(a)). These are thinner than an electrode and designed to sit flat against the mold (Figure 2(f)). When using these to manufacture a clone socket, the electrode indent must, therefore, be backfilled, restoring the shape in this area to match the mold for the original socket (Figure 2(h)). The position of the electrodes needs to be identical for the clone sockets and original, so a plaster replica of the electrode was created via a silicone mold (Figure 2(b)). The depth of the plaster electrode was matched to the difference between the electrode and the dummy. The quality of the molding process meant the plaster electrode could be easily aligned with the original electrode position by lining up the imprints of the metal contacts (Figure 2(c)). The web created by the protective Tegaderm (Figure 2(g)) must also be backfilled (Figure 2(h)). To ensure the infill accurately reflected the original shape, the wet plaster was smoothed to the line of the plaster electrode and existing socket negative using a tongue depressor (Figure 2(d)). The plaster electrode was also colored to help locate the dummy (Figure 2(e)).

### Assessment of clone socket

To assess whether the clone socket accurately replicated the original, 3D scanning was used to compare negative molds taken from each. An Einscan-Pro 3D scanner (Hangzhou Shining 3D Tech Co., Hangzhou, China) was used with “handheld rapid scan” feature and “high quality resolution”; point clouds were generated using “quality priority” and an “un-watertight mesh” created. The scans were aligned using CloudCompare software (v2.12.2), and the distance between them computed using the “cloud-to-mesh” function.

## Results

**Figure 3.**
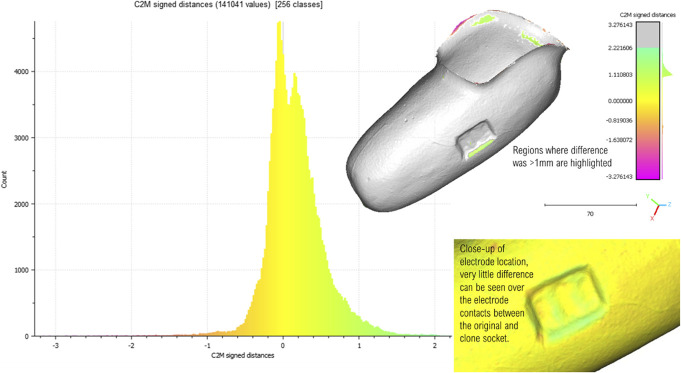
Histogram showing the distribution of C2M distances (in mm) between the mesh of the silicone mold taken from the original socket and the mesh of the silicone mold taken from the clone socket. A positive difference indicates that the surface of the clone lies outside of the original reference scan, while a negative difference indicates the opposite. Top right any distances greater than 1 mm can be seen highlighted on the socket. The discrepancy around the electrodes can be attributed to the Tegaderm web, but as can be seen bottom right, there is almost no discrepancy in the positioning of the electrode contacts. C2M, Cloud to Mesh.

The mean distance between points on the 2 socket scans was 0.16 mm (SD 0.38 mm). A positive difference suggests that the clone socket is volumetrically larger than the original socket. Figure 3 shows a histogram of the distances between the sockets and highlights top right the areas where the difference was greater than 1 mm. There are small areas where the clone is 1–2 mm larger than the original. Around the edge of the electrodes, this was caused by the web created by the Tegaderm not aligning perfectly for each mold. Importantly, the metal electrode contacts show very little error between the 2 molds as shown in the close-up bottom right.

## Discussion

### Accuracy of proposed approach

Our socket cloning technique was shown to be highly accurate. Although it has not been assessed quantitatively, we anticipate this technique will generate a significantly more accurate replica than a new socket manufactured using traditional casting or scanning techniques. We have previously found electrode position alone to vary by several millimeters in different sockets manufactured for the same person, and although not directly comparable, Dickinson et al^8^ present intraprosthetist casting repeatability for lower-limb sockets showing up to 5 mm variation in some regions. Casting and/or scanning and rectification are all processes that rely on the skill of the prosthetist and, for any given socket, would be very difficult to replicate precisely.

### Direct benefits

By removing the requirement for several socket iterations, this technique offers significant time, cost, and labor saving to researchers, technicians, and participants themselves. The process of taking the silicone mold may not be faster than casting due to curing time; however, only the prosthesis is required, thus freeing the participant up to continue with their activities. Most importantly, this process can easily be taken to the participant, without the requirement for a prosthetist. With the confidence that the final socket will fit, it also becomes more feasible to post sockets and molds between labs.^9^

### Broader opportunities

To understand socket-related control or comfort issues, experimental work using someone's own prosthesis or a clone is required. There are several hurdles to using clinically prescribed prostheses for research studies, including risk of damage and complexities surrounding insurance. If researchers wish to explore how the person interacts with their prosthesis, they may want to integrate sensors, which would not be feasible without the involvement of the prescribing clinical team. This socket cloning process would facilitate take-home trials of instrumented prostheses, thus creating opportunities to understand the impact of various factors relating specifically to socket fit. In addition, by manufacturing sockets matching those prescribed clinically, we can assess a range of socket types, originally manufactured by a broad pool of clinicians, rather than by 1 or 2 clinicians affiliated with the research, thereby reducing the risk of unconscious bias. These factors should increase confidence in the validity of results. As our process does not destroy or damage the original negative mold, it also opens new opportunities to evaluate socket design. We could manufacture several sockets with controlled changes, such as adjusting electrode positions or changing wall thickness. Additional electrodes may be added to form arrays, facilitating more applied pattern recognition research. Further, we can explore the impact of manufacturing the socket using emerging materials. By producing these sockets from identical plaster molds, we can reduce potential confounding factors.

### Limitations and recommendations

It can be difficult to remove the initial mold, so experimentation and practice is advised before attempting to clone someone's clinically prescribed socket. We have not yet tested this technique with sockets involving leatherette and would suggest that a thin, flexible, smoother surface is added to aid removal in these cases.

## Conclusions

This case study has demonstrated that our approach to cloning a clinical upper-limb myoelectric prosthetic socket is feasible and accurate (mean difference <0.2 mm). The nondestructive method involves shape capture using silicone and conversion into a plaster mold via alginate. Our method will facilitate the development of research prostheses without the requirement for an on-site prosthetist, creating more opportunities for prosthetics research representative of the clinical realities. In addition, with an increased interest in real-world prosthetics research, this technique will enable engineering-focused labs to develop take-home versions of new technologies allowing exploration of the real-world practicalities of developments in prosthesis control. With funders requiring increased evidence of clinical and cost effectiveness, research must represent the clinical population and their real-world use of their prostheses. This socket cloning technique brings these studies a step closer to feasibility. Although the example socket presented here includes electrodes, this method would be similarly suitable for a nonmyoelectric socket.

## Funding

This work was supported by the UK National Institute for Health Research (NIHR). Award ID: NIHR201310.

## Declaration of conflicting interest

The author(s) disclosed no potential conflicts of interest with respect to the research, authorship, and/or publication of this article.

## Author contributions

AC conceptualized and developed the replication technique. All authors contributed to the development of the methodology for the validation of the technique. AC, JO, and MP developed the methodology for analysis. Funding was sourced by LK and MD. AC undertook the mold production, scanning, and analysis. AC produced the initial draft of the manuscript, and all authors contributed to the subsequent review and editing of the manuscript.

## Supplemental material

Supplemental material for this article is available in this article. Direct URL citation appears in the text and is provided in the HTML and PDF versions of this article on the journal’s Web site (www.POIjournal.org).

## Supplementary Material

SUPPLEMENTARY MATERIAL
