# Supplementary material for: Replication of clinical prosthetic sockets for research purposes
Source: Prosthet Orthot Int. 2024 Nov 27;49(3):353–7. doi: 10.1097/PXR.0000000000000386 (PMC12147737; doi:10.1097/PXR.0000000000000386)
Supplement: SUPPLEMENTARY MATERIAL [file poi-49-353-s001.docx]

**Supplementary material accompanying ‘Replication of clinical prosthetic sockets for research purposes’**

The prosthetic socket fitting process is an artisanal craft performed by prosthetists who use observation and manipulation of the residual limb to understand any anatomical constraints on their design ^[1]^. According to the United Kingdom (UK) Medicines and Healthcare products Regulatory Agency (MHRA), a bespoke prosthetic socket is classified as a custom-made medical device ^[2]^, requiring the instructions of a healthcare professional to be followed in its manufacture ^[3]^. Typically, manufacture involves technicians working from the moulded impression provided by a prosthetist (the healthcare professional).

In the main body of the paper, we report on the proposed method by which clone sockets may be accurately produced without the involvement of a prosthetist, and describe the validation of the method. For the benefit of others wishing to replicate our work, in this supplementary document we explain the techniques we chose not to take forward (and why) (**Figure A1** **– shaded boxes**) together with our recommended technique (**Figure A1** **– white boxes**).


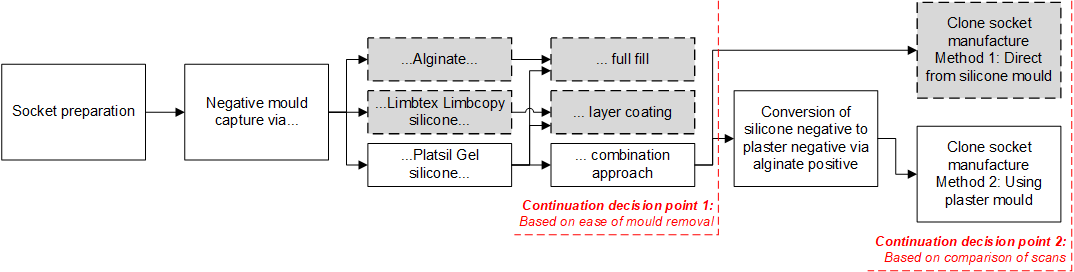


**Figure A1 –** Flowchart showing the stages of socket replication. The white boxes show the process recommended in this paper. The shaded dashed boxes show alternative approaches at each stage which we chose not to take forward.

**Negative mould capture from the original socket**

Limbtex Limbcopy Silicone, Platsil Gel Silicone, and alginate were all evaluated as materials to capture the negative mould from the socket. The Limbtex had a higher shore hardness than the Platsil (shore A-60 for the Limbtex vs A-25 down to 00-30 for the Platsil25 and Platsil00 respectively) and was ~ 50% more expensive. The alginate was considerably cheaper, however, shrinks as it loses water; hence, would need converting immediately to a more permanent form (e.g. plaster). This would not be ideal when replicating the socket in a person’s home. Further, alginate moulds may be damaged during extraction, especially with long, narrow, and awkwardly shaped sockets. We therefore chose to move forward with a silicone moulding technique.

We trialled two approaches to capturing the mould of the socket: full-fill and layer coating.

The **full-fill** technique involved filling the socket with silicone. Even the softest silicone tested (Platsil00), was difficult to extract from the socket. There was not enough compressibility to release the vacuum around the mould and overcome friction between mould and socket. The Limbtex was too hard to remove.

**Layer coating** involved covering the inside of the socket with a layer of silicone. Once cured this was pulled away from the socket. Limbtex held its structure well, however, it was difficult to evenly coat the socket with the uncured silicone. The stiffness of the cured silicone made the thick layers difficult to bend and extract. The Platsil was available with lower shore hardness, making it easier to remove from the socket, however, there was no easy way to ensure the mould did not deform prior to plaster infill.

We therefore took an approach which **combined** the compressibility and ease of extraction associated with the layer coating with the structural benefits of the full-fill. We added a tube of known dimensions into the mould prior to a full-fill (ensuring the tube did not touch the edges of the socket). This could then be removed prior to mould extraction allowing the mould to be compressed and displaced from the socket walls. The tube could then be re-inserted into the mould after extraction to ensure the shape was maintained.

**Manufacture of the clone socket from the negative mould**

We tested two approaches to the clone socket manufacture. Method 2 converted the mould to plaster prior to lamination, as described in the main paper. Method 1 went direct from silicone to the lamination; The details of this approach, which was rejected, are given here.

We initially tested whether the silicone negative (manufactured through the combination approach) could be used directly to make a new laminated socket. We used the 3D scanning assessment technique described above to assess the accuracy of the method. We found a reduction of ~5mm around the supracondylar compressions (**Figure A2 - Left**) between the silicone negative taken from the original socket and the silicone negative taken from the clone socket. This may have been attributed to the vacuum applied during manufacture, potentially combined with the low level of build-up above the trim lines on this mould (**Figure A2 - Right**). We concluded that an alternative technique was needed, leading to the development of Method 2. In addition to increase the size of the build-up above the trim lines and the strength of this region, we increased the size of the tape collar in later iterations allowing us to pour more silicone into the mould.


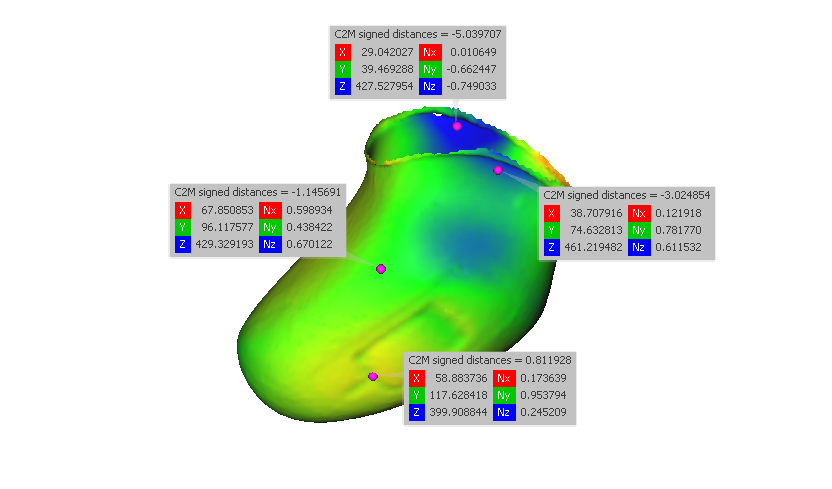


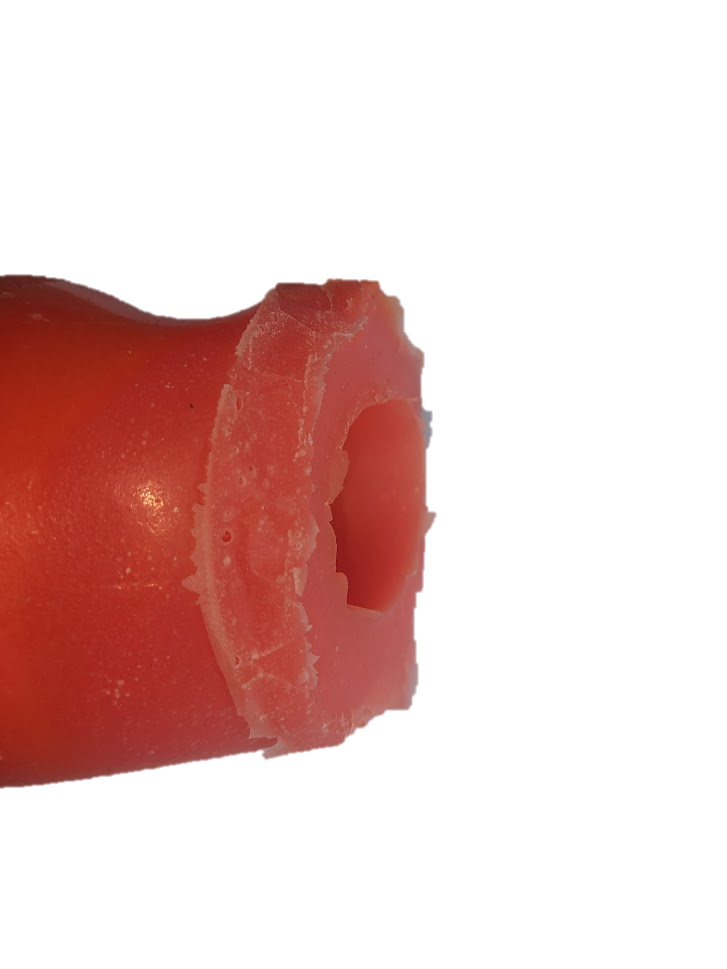


Trimline

Build-up around trimlines

Void filled by tube for ease of mould removal

Socket mould

**Figure A2 – Left:** Difference between the silicone mould of the original socket, and the silicone mould taken from a second socket manufactured directly from the first silicone mould. The colourmap shows the Cloud-To-Mesh (C2M) signed distances between scanned meshes of the two moulds. A reduction in size of ~5mm can be seen in the supracondylar areas (blue), **Right:** Trimline region of original silicone mould showing the build-up amount. A larger build-up may reduce the likelihood of a shape change under vacuum; however, this has not been investigated.

**References**

[1] Olsen J et al. (2022), *The Impact of Limited Prosthetic Socket Documentation: A Researcher Perspective*, **Frontiers in Rehabilitation Sciences**

[2] Guidance on Medical devices: legal requirements for specific medical products – **Gov.uk Medicines and Healthcare products Regulatory Agency website.** <https://www.gov.uk/government/publications/medical-devices-legal-requirements-for-specific-medical-devices/medical-devices-legal-requirements-for-specific-medical-devices> *Accessed 16/08/23*

[3] Guidance on Custom-made devices in Great Britain – **Gov.uk Medicines and Healthcare products Regulatory Agency website.** <https://www.gov.uk/government/publications/custom-made-medical-devices/custom-made-devices-in-great-britain> *Accessed 16/08/23*
